# Formate supplementation enhances folate-dependent nucleotide biosynthesis and prevents spina bifida in a mouse model of folic acid-resistant neural tube defects

**DOI:** 10.1016/j.biochi.2016.02.010

**Published:** 2016-07

**Authors:** Sonia Sudiwala, Sandra C.P. De Castro, Kit-Yi Leung, John T. Brosnan, Margaret E. Brosnan, Kevin Mills, Andrew J. Copp, Nicholas D.E. Greene

**Affiliations:** a Newlife Birth Defects Research Centre and Developmental Biology & Cancer Programme, Institute of Child Health, University College London, London, WC1N 1EH, UK; b Department of Biochemistry, Memorial University of Newfoundland, St John's, NL, A1B3X9, Canada; c Genetics & Genomic Medicine Programme, Institute of Child Health, University College London, London, WC1N 1EH, UK

**Keywords:** Folate one-carbon metabolism, Formate, Neural tube defects, Folic acid, Grainyhead-like 3, mthfd1L

## Abstract

The *curly tail* mouse provides a model for neural tube defects (spina bifida and exencephaly) that are resistant to prevention by folic acid. The major *ct* gene, responsible for spina bifida, corresponds to a hypomorphic allele of *grainyhead-like 3* (*Grhl3*) but the frequency of NTDs is strongly influenced by modifiers in the genetic background. Moreover, exencephaly in the *curly tail* strain is not prevented by reinstatement of *Grhl3* expression. In the current study we found that expression of Mthfd1L, encoding a key component of mitochondrial folate one-carbon metabolism (FOCM), is significantly reduced in *ct/ct* embryos compared to a partially congenic wild-type strain. This expression change is not attributable to regulation by *Grhl3* or the genetic background at the *Mthfd1L* locus. Mitochondrial FOCM provides one-carbon units as formate for FOCM reactions in the cytosol. We found that maternal supplementation with formate prevented NTDs in *curly tail* embryos and also resulted in increased litter size. Analysis of the folate profile of neurulation-stage embryos showed that formate supplementation resulted in an increased proportion of formyl-THF and THF but a reduction in proportion of 5-methyl THF. In contrast, THF decreased and 5-methyl THF was relatively more abundant in the liver of supplemented dams than in controls. In embryos cultured through the period of spinal neurulation, incorporation of labelled thymidine and adenine into genomic DNA was suppressed by supplemental formate, suggesting that de novo folate-dependent biosynthesis of nucleotides (thymidylate and purines) was enhanced. We hypothesise that reduced Mthfd1L expression may contribute to susceptibility to NTDs in the *curly tail* strain and that formate acts as a one-carbon donor to prevent NTDs.

## Highlights

• Neural tube defects in *curly tail* (*ct*/*ct*) embryos are not preventable by folic acid.
• Expression of *Mthfd1L* is diminished in *ct*/*ct* (*Grhl3* hypomorph) embryos.
• Mthfd1L acts in mitochondrial folate one-carbon metabolism to generate formate.
• Supplemental formate reduces the frequency of neural tube defects in *ct*/*ct* embryos.
• Formate alters folate profiles of maternal liver and embryos and enhances folate-dependent nucleotide biosynthesis.

## 1 Introduction

The network of reactions that comprises folate one-carbon metabolism (FOCM) supplies one carbon units for a number of downstream biosynthetic pathways including nucleotide biosynthesis and methylation reactions [1], [2]. Corresponding with the crucial role of FOCM in several cellular functions, abnormal FOCM is associated with a range of diseases, including cancers, fatty liver disease, inborn errors of metabolism, autism, age-related cognitive impairment and birth defects, particularly neural tube defects (NTDs). NTDs, including spina bifida and anencephaly, are a group of birth defects that result from incomplete formation of the neural tube which is the precursor of the brain and spinal cord in the developing embryo [3]. The causes of most NTDs in humans are not well understood owing to their complex etiology which is thought to involve multiple genetic and environmental factors [4]. Sub-optimal maternal folate status is associated with increased risk of an NTD-affected pregnancy while maternal supplementation with folic acid reduces susceptibility [5], although some NTDs are not prevented (‘folic acid-resistant’). Polymorphisms and/or variants in some FOCM-related genes (e.g. *MTHFR*, *MTHFD1L*, *AMT* and *GLDC*) have been associated with NTDs [3], [6], while abnormal thymidylate biosynthesis was identified in a subset of cell lines from NTD patients [7]. Together these data suggest that there is a contribution of abnormal FOCM to NTDs, although it is currently unclear whether this corresponds to NTDs that are preventable by or resistant to folic acid.

Among mouse models of NTDs, some are responsive to folic acid, including *splotch* (*Sp*^*2H*^, *Pax3*) and *Cited2* null strains [8], [9], [10], while others are resistant [11]. Among the latter group, the *curly tail* (*ct*) strain has been studied extensively as a model for human NTDs [12]. Homozygous *ct/ct* embryos develop partially penetrant NTDs comprising spina bifida and/or exencephaly. These defects arise due to failure in completion of neural tube closure in the spinal and cranial regions respectively. Open NTDs only affect a proportion of mutant embryos but even where neural tube closure is completed around 50% of mice develop tail flexion defects owing to delay in spinal closure. The major *ct* gene corresponds to a hypomorphic allele of *Grhl3*, encoding the grainyhead-like 3 transcription factor [13], [14]. However, penetrance of defects is strongly influenced by genetic background [15]. For example, a polymorphic variant of *Lmnb1* was found to influence the frequency of both cranial and spinal NTDs [16].

In addition to genetic modifiers, the frequency of NTDs in the *ct* strain is influenced by several environmental factors, including retinoic acid, inositol and hyperthermia [17], [18], [19]. However, there is no protective effect of folic acid [12], [20]. Supplemental folic acid does not prevent NTDs in *ct/ct* embryos. However, they are sensitive to maternal dietary folate deficiency, which causes a significant increase in the frequency of cranial NTDs and a delay in overall growth and developmental progression, both in *ct* mice and in a genetically matched wild-type strain (+^*ct*^) [21]. The same dietary model does not cause NTDs in other wild-type strains [21], [22], [23], suggesting the presence of predisposing modifier genes in the *ct* genetic background. Analysis of cultured fibroblasts did not indicate a defect of thymidylate biosynthesis in the *ct* strain, but abnormalities of FOCM were observed [24]. For example, the SAM/SAH ratio is lower in *ct/ct* than in +^*ct*^/+^*ct*^ embryos at E10.5, owing to increased abundance of SAH. Moreover, folate-deficiency led to an increase rather than a decrease in SAM/SAH as observed in wild-type and other strains. Diminished methylation appeared unlikely to contribute to NTDs in *ct/ct* embryos as introduction of an *Mthfr* null allele did not increase the frequency of NTDs; no NTDs were observed among *ct/ct*;*Mthfr*^−/−^ embryos [24].

Although FA does not prevent NTDs in *curly tail* mice, we found that intervening downstream of FOCM, at the level of nucleotide biosynthesis may influence the rate of NTDs. Hence, combinations of thymidine and adenine or GMP resulted in a significant protective effect [25]. In the current study we further investigated the potential contribution of altered FOCM to NTDs in the *ct* strain.

## 2 Materials and methods

### 2.1 Mice

*Curly tail* (*Grhl3*^*ct*^) and partially congenic wild-type strains (+^*ct*^*/*+^*ct*^) were maintained as closed random-bred colonies [12], [16]. Wild-type mice for plasma and urine analysis were on a mixed CBA/101 background. The transgenic *curly tail* line (*Grhl3*^*ct*^*/Grhl3*^*ct*^; Tg(*Grhl3*)1Ndeg/0), here denoted *ct/ct*^*TgGrhl3/0*^, carries a BAC that encompasses the *Grhl3* gene, as described previously [13]. Mice carrying a conditional (floxed) allele of *Grhl3* have been described [26]. These mice were crossed to β-actin-*Cre* mice to generate heterozygous null, *Grhl3*^+/−^, mice used in experimental matings in this study. *Curly tail* mice are maintained as a homozygous colony. Other mice and embryos were genotyped by PCR of genomic DNA, as described in the relevant original publications [13], [26].

Animal studies were carried out under regulations of the Animals (Scientific Procedures) Act 1986 of the UK Government, and in accordance with guidance issued by the Medical Research Council, UK in *Responsibility in the Use of Animals for Medical Research* (July 1993).

### 2.2 Supplementation and collection of embryos

Experimental litters were generated by overnight mating. On detection of a copulation plug the following morning, dams were separated and litters designated embryonic day (E) 0.5. Treatments were 20 mg/ml or 30 mg/ml sodium formate in drinking water and control (water only). Doses were based on previous studies [27], [28]. Formate treatment was started from E0.5 and continued until litters were collected, between E10.5 and 12.5. Embryos were dissected from the uterus in Dulbecco's modified Eagle's medium containing 10% foetal calf serum and assessed for the presence of NTDs under a light microscope. Resorptions were recorded and the crown-rump (CR) length measured using an eyepiece graticule. Embryos were rinsed in PBS and stored at −80 °C.

### 2.3 Quantitative real time RT-PCR and sequencing

RNA was isolated using TRIzol (ambion) and DNA removed by DNase ɪ digestion (DNA-Free, ambion). First strand cDNA synthesis was performed using random hexamers (Superscript VILO cDNA synthesis kit) and RT-qPCR was performed with iTaq Universal SYBR Green Supermix (Bio-Rad) on a C1000 Touch Thermal Cycler (Bio-Red). Primers for *Mthfd1l* were: 5′-TCATGGCCGTGCTGGCCTTG-3′ and 5′-TGGCAAAGGGACCAGCGTG-3′ and primers for *Grhl3* were 5′-CCAGACTCCAGTAACAATG-3′ and 5′-AAGGGTGAGCAGGTTCGCTT-3′. Each sample was analysed in triplicate and results were normalised to *Gapdh* mRNA abundance as previously [13], [16]. Similar results were obtained when *Beta-actin* was used for normalisation.

The coding sequencing of *Mthfd1L* was sequenced in *ct/ct* and +^ct^/+^ct^ strains (2 independent embryos for each strain) using cDNA prepared from E10.5 embryos (as above). A series of 8 overlapping primer pairs were used to amplify the entire coding region (28 exons), as well as flanking sequence (100 bp of the 5′ UTR and 210 bp of the 3′ UTR). PCR products were purified using QIAquick PCR Purification Kit (Qiagen). Products were sequenced in forward and reverse directions by Sanger Sequencing (Source Bioscience) and sequences were analysed using Sequencher (GeneCodes Corporation) and compared to the reference Mthfd1L sequence (GenBank XM_006512449.2).

### 2.4 Microsatellite and SNP genotyping

A series of 18 microsatellite markers (D10Mit84, D10Mit80, D10Mit245, D10Mit123, D10Mit306, D10Mit279, RH125020, RH126580, AI265638, 236300, AI317366, AW536662, PMC25853P1), 236299, AU018232, D10Mit49 and 236303) and 4 SNPs (rs47265432, rs249746523, rs218957174, rs217495350) flanking and within *Mthfd1L* on mouse chromosome 10, were tested for polymorphism between genomic DNA of SWR and *curly tail* strains. Polymorphisms were detected either by size difference between microsatellites or by sequencing of PCR products. A synonymous coding SNP rs47265432 was informative. The sequence at this SNP was determined by sequencing of a 432 bp PCR product generated by primer pair 5′-CTCCAGCACTGAGACCCTCT and 5′-TGCTCCACCCTACCTGACTC.

### 2.5 Metabolite analysis

*Folate profile*: Folates were quantified by ultra-pressure liquid chromatography coupled to tandem mass spectrometry (UPLC-MS/MS), as described previously [28], [29]. Briefly, E10.5 embryos were resuspended in ‘folate buffer’ containing 20 mM ammonium acetate, 0.1% ascorbic acid, 0.1% citric acid and 100 mM dithiothreitol at pH 7 and 0.2 μM methotrexate as internal standard. Samples were sonicated using a hand-held sonicator for 10 s at 40% amplitude and an aliquot removed for analysis of protein concentration using the Bradford assay. Protein was precipitated using 2 × volume of acetonitrile and removed after centrifugation (12,000× *g* at 4 °C). Samples were then lyophilised and resuspended in 30 μl ‘folate buffer’. Metabolites were resolved by reversed-phase UPLC (Acquity UPLC BEH C18 column, Waters Corporation, UK) and detected using a XEVO-TQS mass spectrometer (Waters Corporation) operating in negative-ion mode using the following settings: capillary 2.5 kV, source temperature 150 °C, desolvation temperature 600 °C, cone gas flow rate 150 l h^−1^, and desolvation gas flow rate 1200 l h^−1^.

*Formate*: Blood was collected by terminal cardiac exsanguination into lithium-heparin tubes (BD Microtainer) and centrifuged for the isolation of plasma. Blood and urine samples collected from treated mice were taken after 12 days of formate treatment. Formate concentration was determined by gas-phase chromatography mass spectrometry, with urine concentration normalised to creatinine as described previously [28], [30].

### 2.6 Nucleotide incorporation

Incorporation of ^3^H nucleotide precursors into genomic DNA was determined as described previously [25]. Embryos were explanted at E9.5, leaving the yolk sac and ectoplacental cone intact and cultured for 24 h in rat serum containing either [^3^H]-adenine (2 μCi/ml), [^3^H]-thymidine or [^3^H]-CTP (both 1 μCi/ml), with 5 mM sodium formate (formate treated) or an equivalent volume of phosphate buffered saline (Control). Genomic DNA was isolated and incorporation of ^3^H determined by scintillation counting as described previously [25]. DNA concentration was measured by Qubit™ (Thermo Fisher Scientific).

### 2.7 Western blot

The cranial region of embryos at E10.5 was suspended in RIPA buffer and sonicated for 10 s using a hand-held sonicator at 40% amplitude. An aliquot was taken for protein quantitation by Bradford assay. 10 μg of protein was run per sample on NuPAGE 4–12% Bis-Tris gel (Life technologies) and immunoblotted. Blocking was performed overnight with BSA, followed by overnight incubation at 4 °C with rabbit anti-Mthfd1l (1:1000; PA5-31360; Thermo Scientific). After incubation with secondary antibody blots were developed using ECL Prime (GE Healthcare Life Sciences). Blots were stripped and re-probed using mouse anti-Gapdh (1:20,000) and ECL Western Blotting Substrate (Promega). Densitometry was performed using Quantity One software (Bio-Rad).

### 2.8 Statistical analysis

Data are presented as mean ± SEM (n), unless otherwise indicated. Statistics were conducted using GraphPad Prism (version 6.01; GraphPad Software Inc.) and SigmaStat (v3.5, Systat Software). Supplementation data were analysed using Fisher's Exact test. Means were compared using *t*-test or by one-way ANOVA with pairwise analysis by Holm-Sidak test as appropriate.

## 3 Results

### 3.1 Diminished expression of Mthfd1L in curly tail embryos

**Fig. 1:**
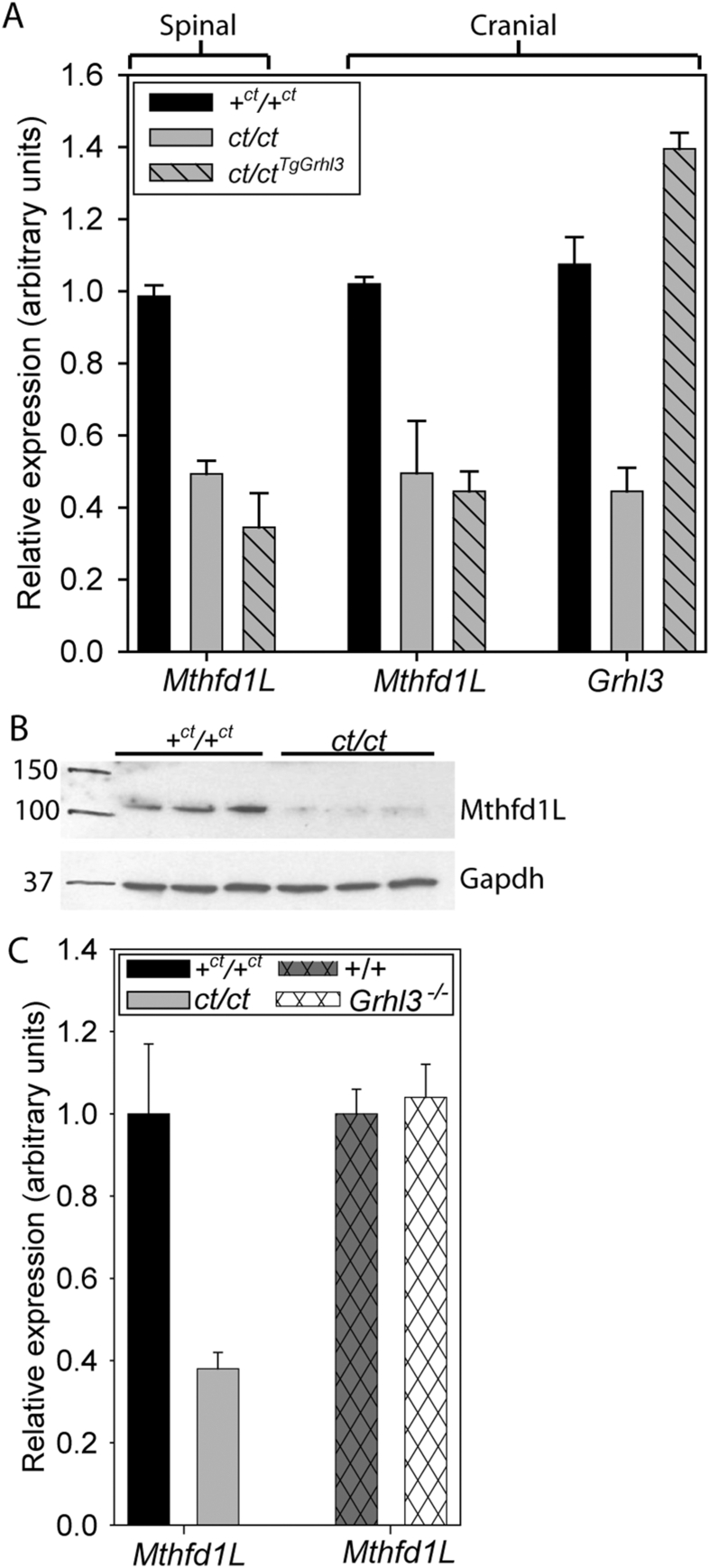
Diminished expression of *Mthfd1L* in *curly tail* embryos. (A) *Mthfd1L* mRNA abundance is significantly lower in the caudal region of E10.5 (28–29 somite stage) *ct/ct* embryos (n = 6) and *ct/ct*^*TgGrhl3*^ (n = 3) than in stage-matched +^*ct*^*/*+^*ct*^ embryos (n = 3; p < 0.001, ANOVA). The cranial region of E9.5 *ct/ct* embryos also exhibits lower abundance of *Mthfd1L* mRNA, which is not corrected in *ct/ct*^*TgGrhl3*^ embryos (n = 2 per group, p < 0.05). In contrast, *Grhl3* expression is diminished in the E9.5 cranial region of *ct/ct* embryos compared with +^*ct*^*/*+^*ct*^ (p < 0.001; ANOVA), and is normalised by the presence of a *Grhl3*-containing BAC (*ct/ct*^*TgGrhl3*^). (B) Immunoblots reveal a 104 kDa band corresponding to Mthfd1L that is less abundant in *ct/ct* samples (caudal region at E10.5) than stage-matched +^*ct*^*/*+^*ct*^ samples. Immunoblot for Gapdh (37 kDa) is used as loading control. (C) Analysis of a second group of E10.5 *ct/ct* (n = 5) and +^*ct*^*/*+^*ct*^ (n = 3) samples replicated the finding of reduced *Mthfd1L* expression in *ct/ct (p < 0.01)*. In contrast, no difference in *Mthfd1L* expression was detected between wild-type (n = 6) and *Grhl3* null (n = 7) embryos.

Curly tail embryos are sensitive to maternal folate deficiency and exhibit some FOCM alterations compared with wild-type [21]. We therefore asked whether *curly tail* embryos exhibited altered expression of genes related to FOCM by interrogating a previously reported microarray study [16], in which mRNA abundance in *ct/ct* neurulation-stage embryos was compared with a partially congenic wild-type strain (+^*ct*^*/*+^*ct*^). Among a panel of 64 genes that encode enzymes related to one-carbon metabolism [31], we noted significant alteration in expression of *Mthfd1L* (2-fold lower in *ct/ct*; p < 0.05). *Mthfd1L* had previously been examined in the context of neural tube closure: SNPs in *MTHFD1L* are associated with NTDs in humans [32], while loss of function of *Mthfd1L* in mice results in cranial NTDs [27]. In order to further investigate *Mthfd1L* expression in *curly tail* embryos we performed qRT-PCR. In samples derived from the caudal region of embryos at E10.5, corresponding to the tissue and stage at which spinal neurulation fails in *curly tail* embryos, *Mthfd1L* expression was approximately 50% lower in *ct/ct* embryos compared with stage-matched controls (Fig. 1A). Expression was similarly lower in the cranial region, analysed at E9.5 which is the stage when neural tube closure occurs at this axial level (Fig. 1A). We further validated this finding by western blot (Fig. 1B). Quantification of band intensity showed a significant reduction in abundance of Mthfd1L protein in *ct/ct* embryos compared with +^*ct*^*/*+^*ct*^ (0.05 ± 0.01 vs 0.43 ± 0.09 arbitrary units, normalised to *Gapdh*; p < 0.01, *t*-test).

### 3.2 Altered Mthfd1L expression in curly tail embryos does not result from Grhl3 deficiency

The main genetic cause of NTDs in *curly tail* embryos is a hypomorphic allele of *Grhl3*. We therefore tested whether *Mthfd1L* may be a direct or indirect transcriptional target of *Grhl3* by analysing expression in *curly tail* embryos carrying a *Grhl3-*BAC transgene [13]. Expression of *Grhl3* was increased in *ct/ct*^*TgGrhl3/0*^ embryos but there was no apparent effect on *Mthfd1L* expression, either in the spinal region at E10.5 or in the cranial region at E9.5 (Fig. 1A). We also analysed *Mthfd1L* expression in *Grhl3* null embryos and found no difference from wild-type (Fig. 1C). These observations suggest that reduced *Mthfd1L* expression in *ct/ct* embryos is not a result of *Grhl3* deficiency. We next considered the possibility that there is an effect of the *ct* genetic background on *Mthfd1L* expression.

The partially congenic +^*ct*^ strain, used for transcriptomic analysis, carries a region of SWR strain DNA at the *Grhl3* locus (and is hence designated wild-type for *Grhl3*). The remaining genetic background is predicted to be 96% identical between *ct/ct* and +^*ct*^/+^*ct*^ embryos, with small regions of SWR DNA remaining in the +^*ct*^/+^*ct*^ strain (as we previously found on chromosome 18 [16]). We tested the possibility that the variation in *Mthfd1L* expression between the two strains results from retention of a region of SWR DNA encompassing the *Mthfd1L* gene in the +^*ct*^ strain. Microsatellite markers and SNPs, flanking and within *Mthfd1L* on mouse chromosome 10, were tested for polymorphism between genomic DNA of SWR and *curly tail* strains. A synonymous coding SNP, rs47265432, was informative; this position is T in the SWR strain but C in *ct* and +^*ct*^. A further intronic SNP rs218957174, typed as T in *ct* and +^*ct*^ but C in SWR. These data indicate that *Mthfd1L* is not in a region where the genetic background of the *ct* and +^*ct*^ strains differs. We also sequenced the entire coding region of *Mthfd1L ct/ct* and +^*ct*^/+^*ct*^ strains and confirmed that there is no difference in sequence between the strains or variation from the reference sequence.

### 3.3 Formate supplementation prevents NTDs in curly tail embryos

**Fig. 2:**
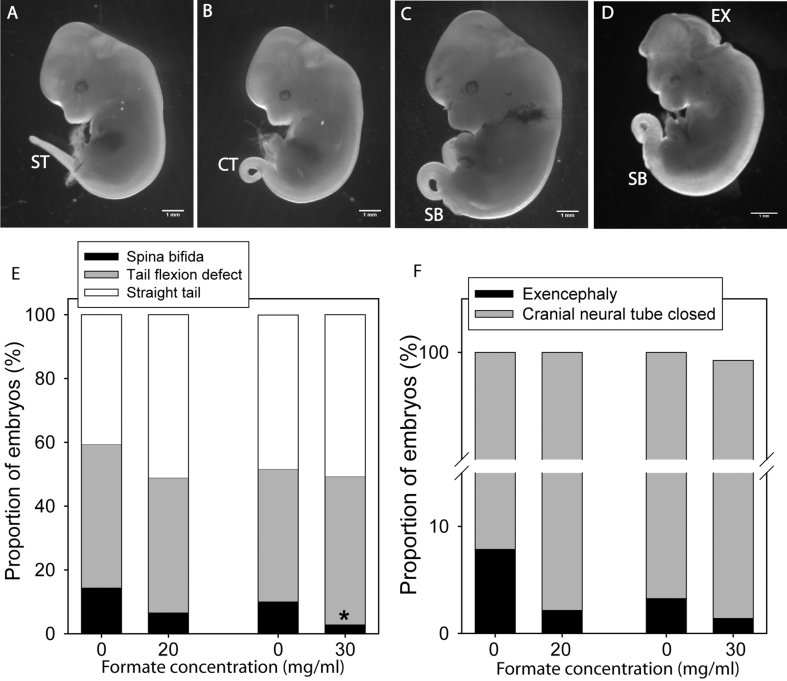
Formate prevents NTDs in *curly tail* mice. Among litters at E12.5 (A–D) we observed embryos with a straight tail (‘normal’, ST), tail flexion defect (curly tail, CT), tail flexion defect with spina bifida (SB) and exencephaly (EX), which can occur in isolation or with a curly tail or spina bifida (scale bars represent 1 mm). (E) Among litters of dams supplemented with formate in drinking water we observed a lower frequency of spina bifida (* indicates significant difference from control group, p < 0.05; n = 187 (0 mg/ml), 90 (20 mg/ml), 91 (0 mg/ml) and 138 (30 mg/ml) embryos). (F) The frequency of exencephaly was lower among all formate-treated litters than among all controls (p < 0.05) although individual groups did not differ significantly (n = 109 (0 mg/ml), 90 (20 mg/ml), 91 (0 mg/ml) and 138 (30 mg/ml) embryos).

Mthfd1L is required for production of formate from 10-formyl THF in mitochondrial FOCM [1]. This is a crucial activity as formate is transferred to the cytosol where it acts as a major one-carbon donor in FOCM. The importance of mitochondrial FOCM in neural tube closure is highlighted by the occurrence of NTDs in mice carrying loss of function alleles of *Mthfd1L*, *Gldc* and *Amt* [27], [28], [33]. We tested whether a deficit of formate production could contribute to *curly tail* NTDs by supplementing pregnant dams with sodium formate in drinking water, as previously used in *Mthfd1L* null mice [27]. Fetuses were scored according to the presence of spina bifida, a tail flexion defect (indicative of delayed spinal neurulation) or a normal straight tail (Fig. 2). Spina bifida occurred in 10–15% of embryos in the control groups. We observed a striking dose-dependent effect on spinal NTDs, with a significant reduction in the frequency of spina bifida, which was present in only 2.9% (4/136) of offspring of dams supplemented with 30 mg/ml (Fig. 2E). The frequency of exencephaly (7.9% and 4.6% in the control groups) also appeared lower in the groups treated with 20 mg/ml or 30 mg/ml formate (2.2% and 1.5%) (Fig. 2F). Overall, exencephaly occurred at significantly lower frequency in formate-treated litters than in control litters (p < 0.05).

We measured crown-rump length of embryos at E11.5 to test whether formate supplementation had an effect on overall growth, but no differences were observed (Table 1). Interestingly however, the litter size among formate-supplemented dams was significantly larger than among contemporaneous control dams (Table 1).

### 3.4 Formate and folate analysis in supplemented dams and fetuses

Quantification of formate by GC-MS confirmed that oral supplementation in drinking water led to a significant elevation in plasma formate in both *ct* and wild-type females (Table 2). The plasma formate level appeared lower in *curly tail* mice than in wild-type, both under normal and supplemented conditions, but this was not statistically significant (Table 2). Nevertheless, we analysed formate in urine to ask whether excretion was higher in *curly tail* females, potentially accounting for lower blood formate. Instead we observed significantly lower formate concentration in urine of these mice (Table 2).

**Fig. 3:**
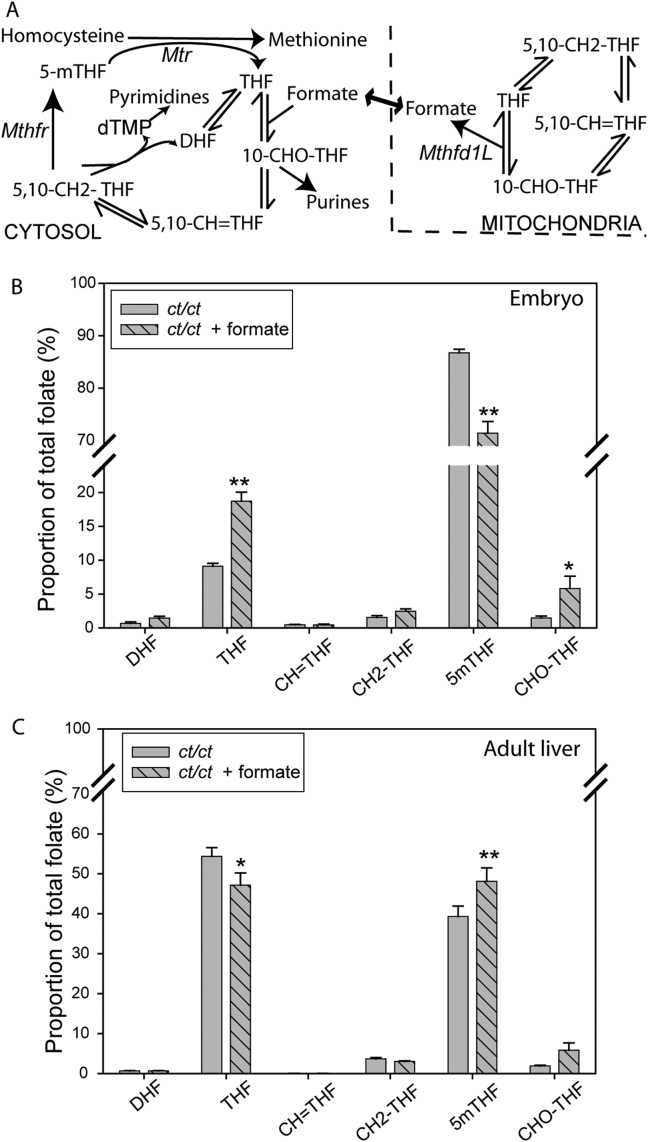
Folate profile of formate-treated *curly tail* embryos and adult liver. (A) Outline diagram of key reactions in FOCM, showing folates analysed. The relative proportions of folates differs in (B) embryos (n = 9 embryos per group) and (C) liver (n = 3) of formate-treated *ct/ct* dams compared with untreated *ct/ct* dams and dams. Data represent the sum of all glutamated forms for each folate. Formate-treated embryos at E10.5 exhibit a significant increase in the relative amount of tetrahydrofolate (THF) and formyl-THF (CHO-THF) and a decrease in the proportion of 5-methyl-THF (5mTHF) (*p < 0.001, **p < 0.0001). In contrast, liver of treated adult mice exhibited a significant increase in proportion of 5mTHF and decrease in THF (*p < 0.05, **p < 0.01). The proportion of dihydrofolate (DHF), methenyl-THF (CH = THF) and methylene-THF (CH2-THF) did not significantly differ with treatment in either embryos or adult liver.

Formate produced by mitochondrial FOCM can enter cytoplasmic FOCM by acting as a one-carbon source for generation of formyl-THF from tetrahydrofolate (THF), mediated by the formyl synthetase activity of the trifunctional enzyme, Mthfd1 (Fig. 3A). We tested whether maternal formate supplementation led to an alteration in the relative proportions of cellular folates using an LC-MS/MS method for quantification of the mono- and polyglutamated (n1-7) forms of seven major folates [28], [29]. In formate supplemented *ct/ct* embryos we noted a reduction in the relative proportion of 5-methyl-THF and an increase in the proportions of THF and formyl-THF (CHO-THF), compared with non-supplemented *ct/ct* controls (Fig. 3B). This was in striking contrast to the liver of supplemented *ct/ct* dams which showed a significant increase in relative proportion 5-methyl-THF and a decrease in THF (Fig. 3C).

### 3.5 Formate suppresses incorporation of exogenous nucleotides into DNA

**Fig. 4:**
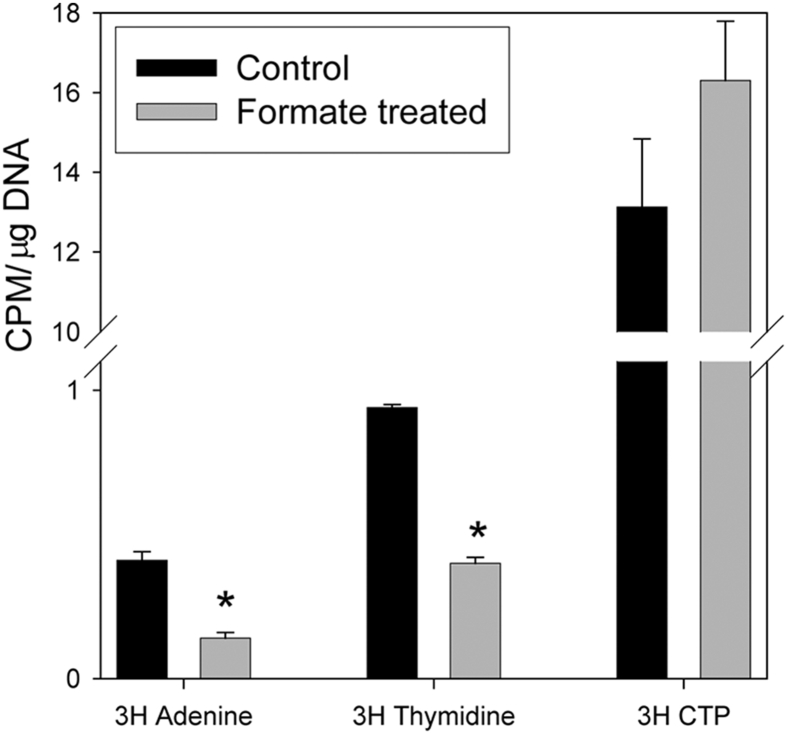
Formate suppresses uptake of exogenous adenine and thymidine. Embryos were cultured in the presence of labelled adenine, thymidine or CTP for 24 h from E9.5. Incorporation of [^3^H]-adenine or thymidine into genomic DNA was significantly lower in the presence of additional formate (p < 0.01). Values are given as mean ± SEM; n = 4–6 embryos per group.

Formyl-THF provides one-carbon units for synthesis of purines while 5,10-methylene-THF is a one-carbon donor for thymidylate (dTMP) synthesis. Alternatively, nucleotide biosynthesis can be mediated through salvage pathways that do not depend on FOCM. For example, thymidine is converted to dTMP through the action of thymidine kinase, while AMP is produced from adenine by the action of APRT. Supplementation of *ct/ct* mice with a combination of thymidine and adenine reduces the frequency of NTDs [25]. We tested whether formate may be utilised in nucleotide biosynthesis by testing its effect on incorporation of exogenous thymidine or adenine in cultured *ct/ct* embryos. Among embryos cultured in the presence of formate we observed significantly lower incorporation of labelled thymidine or adenine (Fig. 4), supporting the hypothesis that formate suppresses the salvage pathways for thymidylate and AMP biosynthesis owing to enhanced use of the endogenous folate-dependent synthetic reactions. Although formate did not affect overall growth we considered the possibility that reduced incorporation of thymidine and adenine could result from decreased DNA synthesis rather than suppression of the salvage pathways. In a control experiment formate did not affect incorporation of labelled CTP into DNA. This finding suggests that overall DNA synthesis was not compromised and is consistent with the fact that FOCM is not required for cytidine biosynthesis.

## 4 Discussion

Spina bifida occurs in 10–15% of *ct/ct* embryos and can be principally attributed to reduced expression of *Grhl3*, being fully rescued by transgenic *Grhl3* expression [13]. In the current study we also observed a significant reduction in *Mthfd1L* expression in *ct/ct* embryos at neurulation stages. Spinal NTDs have been reported in only a very small proportion of *Mthf1dL* null mice [27]. Nevertheless we cannot rule out a potential contribution of reduced *Mthfd1L* expression to spina bifida in the *ct* strain, acting to modify the *Grhl3* effect, as we previously observed for a *Lmnb1* variant (Deletion 18: 56909394) present in the *ct* genetic background. Cranial NTDs (exencephaly) also occur in *Grhl3* null embryos, but only among 2–14% unlike the 100% penetrance of spina bifida [14], [34]. In the current study we found that *Grhl3* expression is less abundant in the cranial region of *ct/ct* embryos than wild-type and is normalised in the *ct/ct*^*TgGrhl3/0*^ embryos. However, unlike spina bifida, cranial NTDs are not prevented in *ct/ct*^*TgGrhl3/0*^ embryos, with exencephaly occurring among around 8% of embryos (n = 140), compared with 6–8% of *ct/ct* embryos (this study and [16]). These findings suggest that cranial NTDs in *ct/ct* embryos depend on genetic factors in addition to the hypomorphic *Grhl3* allele. For example, exencephaly is reduced to 3% among a sub-strain of *ct/ct* embryos with wild-type *Lmnb1* but occurs at 2.6% in a sub-strain of +^*ct*^/+^*ct*^ embryos carrying the *Lmnb1* Deletion 18: 56909394 variant [16]. Both the latter sub-strains exhibit diminished *Mthfd1L* expression similar to the *ct/ct* strain (data not shown). We speculate that this could contribute to cranial NTDs, as observed in *Mthfd1L* null embryos and other loss of function enzymes of mitochondrial FOCM (*Gldc* and *Amt*). In contrast, exencephaly does not occur in the +^*ct*^/+^*ct*^ strain (wild-type for *Grhl3*, *Lmnb1* and *Mthfd1L* expression) unless dietary folate deficiency is imposed [21].

The protective effect of formate supplementation among *ct/ct* embryos supports the hypothesis that there may be a deficiency in supply of one-carbon units from mitochondrial FOCM, as would be predicted in *Mthfd1L* hypomorphs. In addition to reduction in NTD frequency in *Mthfd1L* null embryos [27], formate also rescues NTDs in mice with loss of function of *Gldc* (encoding glycine decarboxylase) [28], another component of mitochondrial FOCM which acts to supply one-carbon units from glycine [1]. Analysis of folate profiles showed that the relative proportion of formyl-THF was increased in supplemented *ct/ct* embryos similar to our previous observations in wild-type or *Gldc* knockout embryos [28]. Interestingly, the proportion of 5-methyl THF declined while that of THF increased in formate-supplemented *ct/ct* embryos compared with controls. This finding may at first appear counter-intuitive. However, this alteration in folate profile was consistent with our previous observation in wild-type embryos [28], whereas formate caused an increase in proportion of 5-methyl-THF and formyl-THF and a decrease in THF in *Gldc* null embryos. In contrast to embryos, in adult liver the relative abundance of 5-methyl-THF was increased by formate treatment while THF declined in abundance. The variation in response to formate supplementation may reflect the observed difference in the baseline folate profiles in these tissues (5-methyl-THF makes up a greater proportion of total folates in the embryo than in liver), which could result from differences in metabolic requirements. In addition, the embryonic response may include secondary effects of altered maternal metabolism combined with increased formate levels.

We previously found that folate deficiency results in a significant reduction in implantations per litter and a significant increase in resorptions in the *ct* strain [21]. Although formate treatment did not affect litter size in our previous study of *Gldc* mutant mice [28], there was an apparent increase in litter size in both cohorts of formate-treated *ct* mice in the current study. This appears unlikely to result from improved survival of post-implantation embryos as there was no difference in resorption rate in control and formate-treated litters (data not shown). Moreover, supplementation was not initiated until the day of finding a copulation plug so it is unlikely that formate affected fertility *per se*. We speculate that formate supplementation may have led to more successful implantations or improved survival of pre-implantation embryos.

How does formate prevent NTDs in the *ct* model? Spinal NTDs in *ct/ct* embryos are known to result from a proliferation defect in the hindgut endoderm, which causes a growth imbalance in the caudal region of the embryo undergoing neurulation [12]. Prevention of NTDs by inositol or by a combination of nucleotides is associated with stimulation of proliferation and normalisation of this growth imbalance [25], [35]. The increased abundance of formyl-THF observed in formate-treated embryos would support supply of one-carbon units for purine biosynthesis. Moreover, suppression of the salvage pathways for dTMP and AMP by exogenous formate supports the hypothesis that supplemental formate can provide one-carbon donors for FOCM-mediated biosynthesis of both purines and thymidylate. This is also consistent with the previous observation that supplementation with combinations of nucleotides (thymidine + adenine or thymidine + GMP) can reduce the frequency of NTDs in *curly tail* mice [25].

Having been found to prevent NTDs in mouse single gene mutants for enzymes of mitochondrial FOCM [27], [28], this study now shows a protective effect of formate in an additional model, *curly tail*, in which NTDs have a more complex genetic basis. The fact that folic acid does not prevent NTDs in this model raises the question of whether formate, or another one-carbon donor, may have therapeutic use in human NTDs. Production of formate has been proposed as one potential mechanism underlying toxicity of excess methanol in humans and primates [36]. Moreover, formate may inhibit the respiratory chain [37]. We did not observe any deleterious effects of 5 mM sodium formate in cultured embryos, although growth retarding and embryotoxic effects of sodium formate have been reported in cultured mouse and rat embryos at concentrations higher than 20 mM [38], [39]. For comparison, *in vivo* formate supplementation (30 mg/ml) of wild-type dams in the current study produced plasma formate concentrations of around 1.2 mM. Nevertheless, rodents may be less prone to formate accumulation than humans owing to a greater capacity to remove formate by oxidation [36]. Moreover, studies in sheep show higher concentrations of formate in the foetal circulation and amniotic fluid compared to maternal plasma [40]. In adult female humans, use of calcium formate at a significantly higher dosage than present in a typical supplement, led to plasma formate concentrations of around 0.5 mM and this was cleared rapidly from the circulation within 4 h [41]. This study was not however conducted during pregnancy and additional safety evaluation would be necessary before considering supplementation in pregnant women. Further studies are justified to investigate the possible value of formate supplements as part of a strategy for NTD prevention alongside folic acid.

## Figures and Tables

**Table 1:** Litter size and embryo size in formate-supplemented *curly tail* mice.

Formate conc.	0 mg/ml	20 mg/ml	0 mg/ml	30 mg/ml
No. Litters	15	10	13	14
Mean litter size	6.90 ± 0.50	9.00 ± 0.90*	7.00 ± 0.60	9.57 ± 0.40**
Mean crown-rump length (mm) (n)	7.78 ± 0.14 (25)	7.683 ± 0.04 (90)	7.88 ± 0.07 (47)	7.78 ± 0.03 (133)

Mean litter size of dams supplemented with 20 mg/ml or 30 mg/ml formate was larger than in control groups (*p < 0.05, **p < 0.01). The crown-rump length of embryos at E12.5 did not differ among experimental groups.

**Table 2:** Formate concentration in plasma and urine ± formate supplementation.

Strain	Plasma formate (μM)		Urine formate (μM/μM creatinine)	
Controls	Formate-treated†	Control	Formate-treated††
*Curly tail* (*ct/ct*)	36.5 ± 3.6	600.3 ± 144.4	15.5 ± 2.8*	438.6 ± 199.6
Wild-type (+/+)	50.7 ± 5.4	1267.0 ± 564.0	47.8 ± 2.9	1884.9 ± 873.5

Formate concentration in urine of female *ct/ct* mice was significantly lower than in urine of wild-type mice (*p < 0.001). Among mice supplemented with 30 mg/ml formate in drinking water, the concentration of formate was significantly higher in plasma (†p < 0.01) and in urine (††p < 0.05) than in the equivalent non-supplemented controls (n = 3–4 mice in each group).
