# Supplementary material for: Formate supplementation enhances folate-dependent nucleotide biosynthesis and prevents spina bifida in a mouse model of folic acid-resistant neural tube defects
Source: Biochimie. 2016 Jul;126:63–70. doi: 10.1016/j.biochi.2016.02.010 (PMC4909716; doi:10.1016/j.biochi.2016.02.010)
Supplement: Supplementary file 1 [file mmc1.docx]

**Supplementary Material - Methods**

Primers used for sequencing *Mthfd1L* cDNA

| Primer name | cDNA region  Transcript: Mthfd1l-003 ENSMUST00000120585 | Primer sequence | Product (bp) |
| --- | --- | --- | --- |
| Mthfd1L_3UTR_F  Mthfd1L_3UTR_R | 5 - 583 | GCTGCTGGCCCTTTACTGT  ATGCACTCTGGGGTCTTCAT | 579 |
| Mthfd1L_F2  Mthfd1L_R8 | 365 - 922 | TCCAAGGAAGTGCTGAGCTT  CACAATGTCTGCCTCTCGAA | 558 |
| Mthfd1L_F6  Mthfd1L_R11 | 730 - 1316 | GGCCAAAGCTGCTGTAGAAC  GGACTTTGGCTTTGCTCTTG | 587 |
| Mthfd1_F11  Mthfd1L_R16 | 1220 - 1797 | CGAGGACAGACTCCAAAAGC  CTCACTTCCTCCTCGGTCAG | 578 |
| Mthfd1L_F14  Mthfd1L_R19 | 1568 - 2126 | CTTCACCTGACTGGGGACAT  TCTGCATCAGATTTGGCTTG | 559 |
| Mthfd1L_F18  Mthfd1L_R24 | 1992 - 2573 | ACATGAAAGAGCGGCTAGGA  TAGCGAGCTCACACACCAAG | 582 |
| Mthfd1L_F22  Mthfd1L_R26 | 2386 - 2969 | TGTAACTGCTGGGGTTCCTC  CGTTCCCACCAAAGGATAAA | 583 |
| Mthfd1L_F24  Mthfd1L_5UTR_R | 2699 - 3290 | GATGTTCAGCTTCCCATCGT  TCTGTGGGTGTGACTGGGTA | 597 |
